# Effects and Influence of External Electric Fields on the Equilibrium Properties of Tautomeric Molecules

**DOI:** 10.3390/molecules28020695

**Published:** 2023-01-10

**Authors:** Ivan Angelov, Lidia Zaharieva, Liudmil Antonov

**Affiliations:** Institute of Electronics, Bulgarian Academy of Sciences, 1784 Sofia, Bulgaria

**Keywords:** oriented electrical field, tautomerism, proton transfer, excited state, transient spectroscopy

## Abstract

In this review, we have attempted to briefly summarize the influence of an external electric field on an assembly of tautomeric molecules and to what experimentally observable effects this interaction can lead to. We have focused more extensively on the influence of an oriented external electric field (OEEF) on excited-state intramolecular proton transfer (ESIPT) from the studies available to date. The possibilities provided by OEEF for regulating several processes and studying physicochemical processes in tautomers have turned this direction into an attractive area of research due to its numerous applications.

## 1. Introduction

The intensity of the external electric field (EEF) can affect ensembles of atoms or molecules, leading to change in a number of fundamental physical and physicochemical phenomena, which can be observed experimentally. For example, splitting of energy levels (Stark effect) in atoms and molecules [1] can be observed spectrally under intensive EEF. EEF causes initialization and activation of the electron [2] and proton transfer [3], changing the geometry of the molecules [4], affecting the interatomic bonds [5] and making possible chemical interactions [6,7,8]. Fried et al. [2] in 2017 defined several basic principles regarding the ability of electric fields to play an important role in the enzyme catalysis. The most important, which can be extended to chemical processes in general, are listed below [2]:

- noncovalent interactions between a given molecule and its local environment (including H-bonds) can be expressed and quantified in terms of the electric field, which the environment exerts on the molecule;
- the electric field created by the environment can be experimentally measured through the vibrational Stark effect, which maps the frequencies of vibrational probes to the electric field experienced by that vibration;
- a chemical reaction can be catalyzed by an electric field if the reactant’s charge configuration (dipole moment) changes upon passing to a transition state. If the dipole moment increases (decreases) in magnitude, an electric field of greater (smaller) magnitude will accelerate the reaction; if the dipole reorients, an electric field aligned with the transition state’s dipole orientation will accelerate the reaction.

As noted by Gorin et al. [9], the chemists most often focus on the use of catalysts and their electronic properties, to lower the barriers in the desired reaction. A complementary strategy is to change the medium (solvent) in which the reaction takes place. There is very little information about the possibility of controlling the local electrostatic environment in order to achieve the desired response. At the same time, a strong electric field can, in principle, affect the reaction through the interaction electric field—dipole moments of the catalyst, reagents, or solvent molecules, making it possible to significantly reduce the respective reaction barriers. Therefore, if the internal electric fields can be modulated by adjusting the parameters of the applied external electric field (orientation, value), these effects can be widely used to carry out selective chemical processes. The recent book Effects of Electric Fields on Structure and Reactivity: New Horizons in Chemistry, edited by S. Shaik and T. Stuyver [10], testifies to the role of EEF in modern chemistry. The basic theory is introduced and the effects of electric fields on structure and reactivity are demonstrated, encouraging new applications in science and technology.

Tautomerism is one of the chemical processes of isomerization with great importance in biology, drug design and technology [11,12], where the use of EEF could bring substantial advantages. Tautomeric forms *(enol-* and *keto-* as a general definition [12]) differ by the position of a proton in the structure of a given organic molecule. The process is strongly influenced by the local environment: solvent, acidity, UV-irradiation, temperature and addition of metal ions. The change of the solvent is one of the most often used strategies to shift the tautomeric process in one or another direction. At the same time such a procedure is extremely technologically inconvenient, especially in potentially high-tech applications in molecular electronics and machinery. Bearing in mind that the solvent effect is substantially based on the different stabilization of the tautomeric forms due to their different dipole moments (implicit solvation), the use of EEF can give another option to control the tautomeric state in a single solvent (i.e., without changing the solvent).

Therefore, in this review paper, we attempt to briefly summarize the influence of the external electric field on an assembly of tautomeric molecules and to what experimentally observable effects this interaction can lead to. The influence of EEF on the proton transfer is the focus of our interest. The information available to date about the influence of an OEEF on the excited-state intramolecular proton transfer (ESIPT) [13] will be especially discussed, due to the importance of this process in science and technology in the last decades. It comes from the conceptual idea that a single organic molecule could play the role of an electronic device [14], which leads to design of molecular wires [15,16,17], molecular rectifiers and diodes [18,19], molecular information storage devices [20,21], molecular switches [22,23,24] and molecular logic switches [25]. All these examples indicate the importance of the good understanding of the phenomena of the EEF influence on the processes in molecular systems.

## 2. Photophysics of Reactions Induced by External Electric Fields

The concept of interaction management when applying an external electric field is studied by means of theoretical calculations. Applying the density functional theory (DFT), it is concluded that constant electric fields in the range of 1–10 V/nm change the barriers of activation of gas-phase reactions by several kcal/mol. These values are sufficient to change the selectivity of reactions with several orders of magnitude at room temperature [26,27,28]. On the other hand, the application of large electric fields on reaction volumes is a challenge because when they are applied only at short distances it leads to dielectric failure and breakthroughs in the medium. In [29], the fields in the range of 0.01–0.1 V/nm applied to thin (μm size) frozen samples affect the rates of electronic transfer reactions due to the significant proportional changes in the dipole moments of reactants [30,31].

Only properly and reliably registered processes, taking place in a reaction environment [29], are needed to prove the EEF influence. At the present stage of development of the research in this field, it is assumed that the most reliable information can be obtained by using spectroscopy. This is due to the fact that the effect of the applied electric field on an atomic or molecular system leads to a change in the absorption or emission spectrum of this system. The manifestation of the interaction of EEF with atoms and molecules is known as the Stark effect. The terms electrochromism, electroabsorption and electrooptical absorption have also been used in the literature to describe the same phenomenon. The change in the frequency **∆*ν*** of the molecular transition in the presence of an externally applied electrostatic field $\vec{F}$ is given by the following equation [1,29]:

(1) $$\Delta \nu =-\Delta \vec{\mu }\cdot \vec{F}-\frac{1}{2}F\cdot \underline{\Delta \alpha }\cdot \vec{F}$$

where **∆*µ*** and **∆*α*** are the changes in the dipole moment and the polarizability between the initial and final energy state, respectively. These two terms of (1) are also known as linear and quadratic Stark effects, respectively, because **∆*μ*** results in a frequency shift linear in $\left|\vec{F}\right|$. At the same time, for a transition in an uniaxially oriented system, the influence of **∆*α*** leads to a shift quadratic in $\left|\vec{F}\right|$. It should be noted that such uniaxial orientation of the reaction system, although highly desirable, is rarely achieved in the practice, except in simple systems.

**Figure 1:**
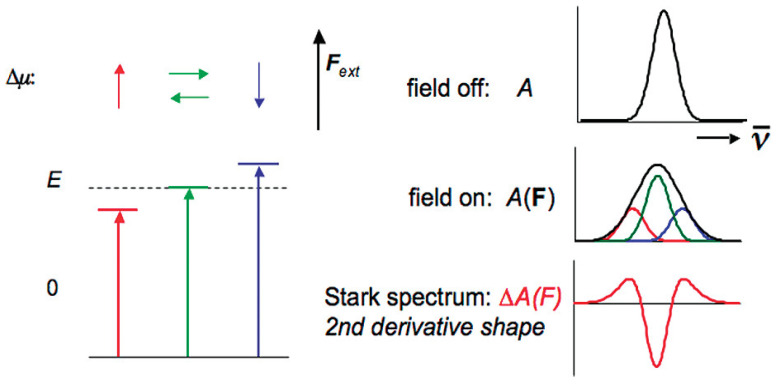
Scheme of the shape of the Stark spectrum line of an isotropic, stationary sample with a dominant contribution of $\Delta \vec{\mu }$. In red, blue and green are shown vectors corresponding to **∆*µ*** parallel, antiparallel and perpendicular to the applied field ${\vec{F}}_{ext}$. The energy shift, in the absence of an EEF, is shown by the following equation: $\Delta E=-\Delta \vec{\mu }\cdot {\vec{F}}_{\mathrm{ext}}$. The consequence for the spectrum is shown on the right: some orientational subpopulations are shifted to lower energy, some to higher energy and some remain about the same. The broadened spectrum is the result of the difference between the field-applied spectrum and the fieldless spectrum, which has the second derivative line shape. Reprinted with permission from [30]: Copyright 2009 American Chemical Society.

The simplified explanation in physical terms is that the random orientations of Δμ in relation to the direction of the applied EEF lead to changes in the energy levels of the transition state and, accordingly, to broadening the spectrum of the transition. Mathematical modelling of the Stark effect shows a spectral shape, similar to a second derivative curve (Figure 1), which results from the difference between the filed-on and field-off spectrum. When interacting with the field, the molecular polarizability **∆*α*** changes, causing a dipole moment, usually in the direction of the field. Since this field-induced dipole moment is field-oriented, the spectrum shifts to either higher or lower energy, depending on the sign of Δα. This shift gives a shape, which could be associated with the first derivative of the absorption spectrum. The origin of these observed effects with respect to the absorption spectra can be understood by considering a hypothetical example of a molecular ensemble characterized only by two energy levels. The influence of the EEF applied parallel or in the opposite direction of the dipole moment Δμ is shown in Figure 1.

In [32] the authors show that when an EF is applied to a molecule, the magnitude of each energy level shift (Stark effect) depends on the electric dipole moment (μ) and molecular polarizability (α) of the concerned state. For an isotropic and immobilized sample, application of an external electric field will broaden the optical spectra due to a change in μ following optical transition. This change gives rise to a Stark effect line shape, which is given by the second derivative of the field-free absorption or emission spectrum. However, if the molecular polarizability varies following optical transition, the Stark effect line shape corresponds to the first derivative of the field-free spectrum.

The following aspects of the Stark effect are fundamentally relevant:

- classical Stark effects—applications in spectroscopy for experimental determination of the change of the dipole moment **∆*µ*** and the polarizability **∆*α*** for a given transition;
- non-classical Stark effect—the influence of the electric field on the dynamics of processes or populations of energy levels in chemical reactions.

**Figure 2:**
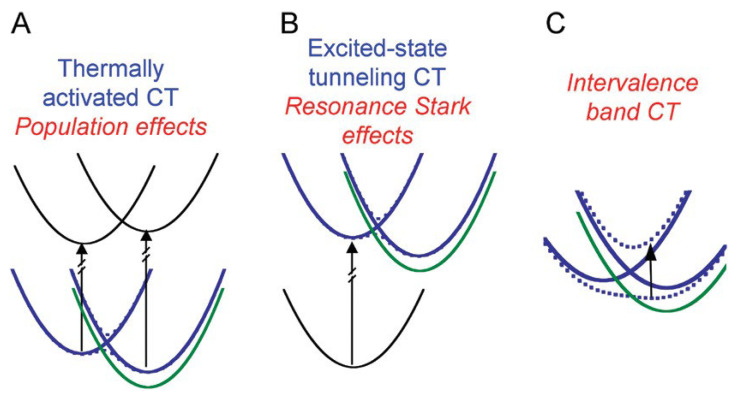
Scheme of the energy level diagrams showing three types of the so-called nonclassical Stark effects in which the absorption or emission line shape is affected. All levels in blue correspond to absence of the applied field, while in green the shift in level for orientation of the field is given. (**A**). Application of the field, for population effects, shifts one of the states to lower energy and so the populations are supposed to respond as the system returns to equilibrium. (**B**) A similar effect is expected to occur for excited-state electron transfer. The rate of electron transfer is changed as the charge-separated-state energy is shifted by the field change. Because of the mixing of the locally excited and charge-separated state, the ground-state is also affected by the electric fields, which leads to “resonance Stark effects”. (**C**) The direct transfer of an electron from one part of a molecule to another corresponds to intervalence charge transfer bands. Theoretical investigations, such as [33,34], can be used as a description for the intensity, position, and line width of these transitions. It is expected that an application of a field will shift the relative energy of the states involved in the transition and will lead to intervalence band Stark effect line shapes. Reprinted with permission from [30]: Copyright 2009 American Chemical Society.

Boxer et al. [29,30] explained how an electric field can perturb the populations or reaction dynamics of processes (nonclassical Stark effects). Dynamics are affected in such a way that the resulting line shapes are different from the simple sums of derivatives described for classical Stark spectroscopy. Three types of nonclassical Stark effects, in which the absorption or emission line shape is affected, are shown in Figure 2.

Nonclassical Stark effects occur naturally in systems, where charges, under the influence of EEF, are sensitively displaced, such as electron and proton transfer reactions, and where this can be spectrally detected. Therefore, we might consider the tautomerization process as a nonclassical Stark effect, where the resulting electric field, due to applied EEF or the solvent’s orientation of the dipole moments, plays the role of an oriented electric field.

In principle, the field ***F***, at each point of the space,

(2) $$F=F_{int}+f\cdot F_{ext\;}{\;}_{\;}$$

can be approximated as the sum of two fields: one due to the dipole matrix $F_{\mathrm{int}}$ and second, a term that is proportional to the external field $F_{\mathrm{ext}}$ (2). $F_{\mathrm{int}}$, is the local field in the absence of an applied field, which considers the solvent reaction environment [30,31,35] and/or the effects due to another organized local structure. $F_{\mathrm{ext}}$ is the mean field, external to the investigated sample molecules in the nearby solvent molecules. The external field is equal to the applied field, which is the applied voltage U divided by the distance d between the electrodes by means of the expression $F_{\mathrm{ext}}=U/d$. The correction factor f of the external field is a tensor (in a general case), which is usually approximated by a scalar quantity for simplicity (in the range 1–4). In addition, the inhomogeneity of f within the chromophore volume and the variability of f between different chromophore molecules are usually ignored.

Shaik et al. in [36] make an overview of how to design and apply an external electric field in order to obtain effects on bonds, structures and reactions. Examples of bond breaking, reactivity/selectivity control and OEEF-initiated mechanisms in chemical structures are described.

In order to assess the effect of the OEEF on experimental strategies for investigation of the influence of electric field in chemical reactions and synthesis [37,38], the authors have tried to demonstrate the potentially high impact in case of skillful exploitation of electric fields. They give an overview of some of the recent theoretical progress made towards a more fundamental understandings of the effects of electric fields on chemical reactivity and conformation changes. Among the others, they remark that EEFs have been demonstrated to be capable of inducing catalysis, inhibition, bond dissociation, regio- and stereo-selectivity, spin-selectivity and so forth. The general conclusion is that the eventual blossoming of the field of research of EEF influences in physicochemistry seems almost inevitable.

Because the internal Stark effect changes the energies of electronic excited states, its action cannot be limited to the shifts of absorption and emission spectra only. The electrostatic fields can dramatically modulate the probabilities and rates of excited-state reactions. This fact is manifested clearly in the operation of the photosynthetic reaction center, where the primary charge separation in the symmetric special pair, instead of being random, achieves unidirectionality under the influence of the electrostatic field [39,40]. There are other examples of the influence of electric field on excited-state reactions in the electron transfer [41,42] and formation of excimers and exciplexes [43]. The corresponding studies on excited-state intramolecular proton transfer (ESIPT) systems in the period before 1990 are lacking. The ESIPT process [3] generally involves transfer of a hydroxyl (or amino) proton to a proton acceptor such as a carbonyl oxygen or a nitrogen atom in the excited state, resulting in a large Stokes shifted tautomeric emission. Initially, this relation was observed in the solvatochromic studies [44,45,46]. Bearing in mind that solvatochromic and electrochromic effects are intrinsically connected [47], one should expect strong electric field influence on the ESIPT reaction in this system. In this respect, 3-hydroxyflavones (3-HF) are of special interest, because the excited-state proton transfer in this system does not result in charge separation. Furthermore, it occurs in the direction of compensation of charge asymmetry created in the normal excited state [48,49]. Because the ultrafast proton tunneling is a key elementary step of this reaction [50,51,52,53], the relative contribution of the excited normal (N*) and tautomeric (T*) forms [13] in the emission should be determined by energetic factors. This is probably the origin of the good correlation between spectral shifts and changes in $I_{N^{*}}/I_{T^{*}}$ (emission spectra intensity ratio) that was observed in the experiments. We can refer to [54], as a validation of this theory, where it is shown that the internal Stark effect (internal electrochromy) is in the origin of the very strong internal electrochromic modulation of ESIPT in 3-HF. Fluorescence spectra of 3-HF derivatives with charged groups attached to the chromophore from the opposite sides without ***π***-electronic conjugation were compared with those of their neutral analogues in a series of representative solvents. The introduction of the proximal charge results in shifts of the absorption spectrum and of both N* and T* emission bands, which correspond to initial and phototautomer states of the ESIPT reaction. The observed shifts (in the range from 500 to 1100 cm^−1^) are in accordance with the Stark effect theory.

It is shown that the electrochromic shifts of spectra are the result of interaction of the chromophore ground- and excited-state dipoles with electric field produced by the proximal charge [47]. In the simplest dipole approximation, the direction and magnitude of the shift $\Delta \nu_{\mathrm{obs}}$ [55] are proportional to the change of dipole moment associated with the spectroscopic transition $\Delta \vec{\mu }$ and the electric field vector $\vec{F}$:

(3) $$h\Delta \nu_{obs}=-\left(\frac{1}{\varepsilon_{ef}}\right)\;\left|\Delta \vec{\mu }\right|\;\left|\vec{F}\right|\;\cos\theta \;$$

where θ is the angle between the $\Delta \vec{\mu }$ and $\vec{F}$ vectors, and ***ε_ef_*** is the coefficient that accounts for dielectric screening, being a microscopic analogue of dielectric constant ***ε***. It was shown [47] that changes in optical absorption due to the action of an external electric field are mainly due to the following three effects: orientation effect, band shift effects and direct field dependence of the transition moment. Therefore, in the experiments, the changes (including tautomerism related ones) in the absorption spectrum of a molecule in an external electric field result from the superposition of these three effects, and a full theoretical description leads to relations that can reveal the contribution of each one.

An important feature in proton transfer reaction mechanism is the coupling between the proton motion and the electron density redistribution known as Proton Coupled Electron Transfer (PCET) [56,57]. The electrostatic stabilization is the main reason for such reactions. The results are as follows—the electrons can be driven to species where they are needed, or the protons can be delivered to a site where they are needed with the help of collateral proton/electron transfer. In general terms, the reason for this coupling is simple—positive protons and negative electrons are driven to each other. However, there are some cases, such as cytochrome oxidase (CcO), where electrons push the protons against the electric field (EF) gradient [58]. Cases like this one are those which uncover some of the most interesting effects of the field to the so-called coupled transport, and questions of influence of EEF on this process are important.

The prospects in the theoretical study of PCET are exciting and challenging. Based on the significance of designing solar cells, which often involve PCET at a fundamental level, the development of methods to experimental study of the photoinduced PCET reactions is critical. On the other hand, not enough experimental investigations for PCET on this moment are published.

Che et al. [59] discussed, from their perspective, the importance of taking electric fields into account when modeling catalytic systems. They explain that the field can influence electronic interactions, such as adsorption structures, vibrational frequencies and oxidation states, in adsorbate/metal systems, and alter thermodynamic and kinetic properties of elementary reactions. The presence of an electric field may alter the reactivity and selectivity in any polarizable system, such as methanol synthesis, reactions involving water, reactions with aromatics and metal/metal oxide supports.

Ciampi et al. [60] review the catalysis of single molecules and in electrochemical and chemical systems in the presence of EEF. Three broad experimental platforms are described, in which the implementation of electrostatic effects in chemistry is successful. An outlook of the wide-ranging potential for use of external electric fields for controlling chemical reactivity and selectivity is provided.

At a single molecule level, OEEFs can be generated within scanning tunneling microscope (STM) experiments. By attaching reagents to the tip and substrate of the STM, and operating in either blinking or tapping mode, these experiments can deliver an OEEF while measuring the effect of its strength and bias on the reaction rate. Consequently, chemical reactions on the base of induced electron tunneling can occur. Other types of realized reactions are on the base of conditions for bond forming or for bond breaking, which accelerate or decrease reaction rates.

**Figure 3:**
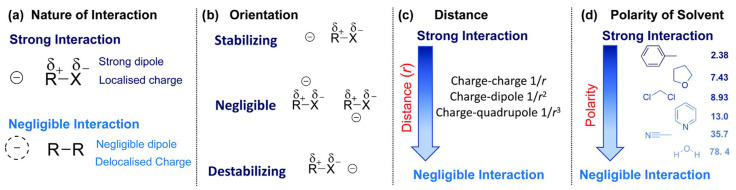
Some factors that affect the strength of electrostatic effects on the stability of a species R–X, including the nature of the interaction (**a**), the orientation of the charge respect to the bond dipole (**b**), the distance of the charge from the bond (**c**) and the polarity of the reaction medium (**d**) as quantified by its dielectric constant. Used with permission of Royal Society of Chemistry, from [60]: permission conveyed through Copyright Clearance Center, Inc.

In Figure 3, factors which influence the strength of impact of EF on investigated interactions are presented.

It should be noted that these factors reflect the impact of a charge on the stability of an isolated species. To assess the impact of the charge on a reaction barrier or enthalpy, one needs such charge effects to change over the course of a reaction so that they do not cancel from the barrier and/or reaction enthalpy. As seen in the figure above, a major role in the magnitude of the electrostatic effect is played by the nature of the interaction and the alignment of the charge. Best effects are observed when the alignment of the charge is along the bond axis, and the worst are observed when the alignment is orthogonal to it. According to the sign of the charge, or upon which side of the bond it is placed, it can be said whether the effect is stabilizing or destabilizing, depending on polarizability. If there is a polarizability the species is more resonance stabilized and therefore the stabilization is larger. The charge group effects on radical stability correlate inversely with the spin density on the normal radical center within a homologous series, and they also correlate positively with the acid dissociation constant (**p*K*_a_**) of the acid [61]. As shown in the figure, there are also other important aspects which should be considered. For example, the charge-group effects on stability decay with the distance in accordance with Coulomb’s law. The charge dipole interactions decay as $1/r^{2}$ and a charge quadrupole interaction as $1/r^{3}$, where r is the distance from the charge to the bond. They also depend on the polarity of the reaction medium. If the effect of a charged group on a reaction barrier or enthalpy is of interest, it is important for the effect of the charge to differ over the course of the reaction so that it does not cancel.

## 3. Influence of Oriented External Electrical Fields (OEEF) on Tautomeric Molecules

**Figure 4:**
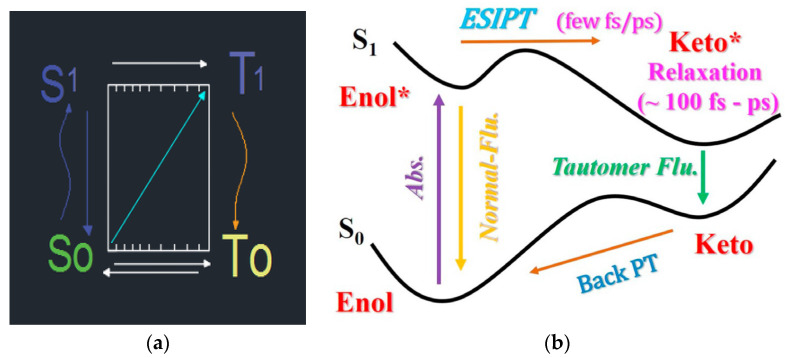
Sketch of a tautomer process (**a**) with contribution of ground and excited states (S_0_, S_1_, T_0_ and T_1_ represent ground and excited states of enol- and keto- forms, respectively); a closed four-level-cycle process of proton transfer (**b**) of a tautomeric compound. Reprinted from [66]: Copyright (2020), with permission from Elsevier.

Tautomerism is a phenomenon in which a chemical compound tends to exist in two or more interconvertible structures that differ in the relative position of one of the atomic nuclei, which is usually hydrogen (a proton, prototropic tautomerism) [12]. Depending on the relative stability, tautomers might exist either as single tautomer or as tautomeric mixture. Most specifically, tautomerism involves a change in the position of the hydrogen with an associated change in the position of the double bonds. Since such systems can exchange the proton in ground and excited state [62], as sketched in Figure 4a, the efficiency of the excited state tautomerization process is an important parameter, crucial in the design of fluorescent markers used in biological research [63,64,65]. Ground and excited tautomeric transitions are sketched in Figure 4b along with the ground ($S_{0}$) and single excited ($S_{1}$) energy surfaces of the tautomers. So-called four-level-cycle process in which target molecules undergo Enol → Enol* → Keto* → Keto conversion is shown. According to the number of protons transferred, the ESPT is divided into two types—excited state single proton transfer (ESSPT) and excited state multiple proton transfer (ESMPT). It should be remarked that reaction in a single-molecule framework uses excited state intra-molecule proton transfer, whereas a reaction involving two or more groups uses excited state inter-molecule proton transfer. Consequently, the spectroscopic investigations of tautomers in the presence of EEF are related to the transition of the compound in excited energy state, where ESIPT exists. According to Dong et al. [66], a variety of new chromophores have been designed to exploit the properties of ESIPT. It is of extreme importance to clarify the mechanisms of this transfer, exploring all kinds of conditions in which it occurs. The hydrogen bond (HB), which is usually expressed as X–H ⋯Y, where X represents the proton donor (usually either O or N) and Y represents the proton acceptor (O, N, or C in other groups), is considered as a precondition for ESIPT. In the same review the mechanism of ESIPT in complex environments is discussed, including solvents of different polarity [67,68] and the presence of an external electric field [69]. Main attention is given to the modulating environmental effects on the ESIPT reaction, such as increased polarity, direction or force on the electric field or the concentration of ions added to the solution [70].

Steady-state and time-resolved techniques are crucial for any experimental investigation of ESPT. The most effective tools for studying ESPT are pico-second kinetics, femto-second time-resolved spectroscopy as well as vibrational time resolved spectroscopy [12]. However, it is difficult to reveal the dynamic evolution in detail by correlation between the geometrical configurations and spectra based on the experimental results. Therefore, it is of a great importance to investigate ESPT at a molecular level. However, such studies are very expensive, due to the need of special instruments, such as scanning tunnelling microscope, equipment for femto-second transient absorption measurement etc. Simplified, the electron can be raised from the ground to an excited state during photostimulation, leading to a change in the charge density around the molecules and resulting in a redistribution of charge in and around the molecules. This makes the proton donor more acidic and the proton acceptor more basic. The destabilization of the HB force gives an impulse for ESPT, leading to the formation of an unstable zwitterionic tautomer, which in the process of de-excitation eventually returns to its original configuration due to Coulombic interactions [70,71,72]. The experimental observation of the transfer from Enol* to Keto* form is accompanied by experimental registration of emission with a large Stokes shift—up to 7000–10,000 cm^−1^ [73,74]. The process is very fast (femto-second time scale) and very sensitive to the environment [75].

The question that immediately arises is how the EEF will affect this phenomenon in real systems.

The brief review in [13] reveals various practical aspects of ESIPT phenomenon, and as the authors outline, the key issue is to assess the essential aspects regarding proton transfer dynamics. For this reason, the influence of EEF on the fast and slow ESIPT, on the irreversible and reversible ESIPT, on the hydrogen bonding at the excited-state double proton transfer (ESDPT), on the excitation wavelength and on other phenomena related to the tautomerism is of substantial interest. In general, the efficiency of the tautomerization can be estimated from the excitation spectra. For example, in the case of ESIPT of azo dyes and Schiff bases, depending on the ground state relative stability, the excitation spectrum has contribution from both *enol*-like (E) and *keto*-like (K) forms with emission coming only from the *K* tautomer. In this case, the efficiency (η) can be expressed by [76]:

(4) $$\eta =\left(I_{exc}\left(E\right).A_{K}/I_{exc}\;\left(K\right).A_{E}\right)\;$$

where $A_{E}$ and $A_{K}$ are the measured absorbances at the maxima of ***E*** and ***K*** forms, respectively, $I_{\mathrm{exc}}\left(E\right)$ and $I_{\mathrm{exc}}\;\left(K\right)$ are the excitation intensities at the same wavelengths. This approach can be adopted to estimate the influence on efficiently of the ground and excited-state tautomerization, where the respective excitation intensities and absorbances are being replaced with these at or without applied EF.

As we noted above, the EEF affects the tautomeric processes, i.e., proton transfer and double bond reorganization in the tautomeric compounds, in both the ground and excited states. At the same time, the study of these processes is carried out in the presence of solvents, characterized by different polarity, dipole moments and proton donor and proton acceptor abilities. All these properties of the solvent should be affected to one or another extent by the presence of EEF. Therefore, the effects of EEF on the tautomeric equilibrium need to be considered only through the interaction of three complex systems—the individual tautomers, being of different polarity, located in the solvent; the molecules of the solvent interacting by means of their dipole moments either with the tautomers or with each other; and the nature of EEF—stationary or pulsed with the corresponding orientation. The orientation of the stationary EEF should be with respect to the dipole moments of the solution, while the pulsed EEF should be oriented with respect to the direction of translation of the proton (electron) or the dipole moment of the tautomeric molecule.

In [77], the authors using molecular dynamic (MD) simulations demonstrate the degree of solvent influence when EEFs are applied on an investigated system. They have found that the EEF causes the solvent re-organization; the solvent molecules dipole moments gradually align with the applied field as the field strength increases. The collective orientation of the solvent molecules modifies the electrostatic environment around the investigated compounds and induces a global electric field pointing in the opposite direction to the applied EEF. The combination of these two interrelated effects results in a partial or complete shielding of the EEF, with the degree of quenching being proportional to the polarity/polarizability of the solvent. Nevertheless, the authors find that the beginning of the investigated reaction (in this case Menshutkin reaction catalysis) inevitably emerges once the value of EEF exceeds value of the induced opposing field of the solvent. The main idea is that the electric fields impart velocity on catalysis by disproportionally stabilizing the so-called “valence bond charge transfer states (CTS)”, which are responsible for the charge flow from one reactant to the other and thus for the emergence of polarization within the reaction complex. The authors remark that these CTS states do not contribute in an equal way to all the species along curve of the potential energy surface (PES). Some stationary points are more affected by them than others, and this differential susceptibility along the PES is what causes the catalysis, reducing the global barrier associated with the process. The authors conclude that in order to assess the effect of the OEEF, the reactant system should be situated in a point with low energy barrier, corresponding to the PES.

**Figure 5:**
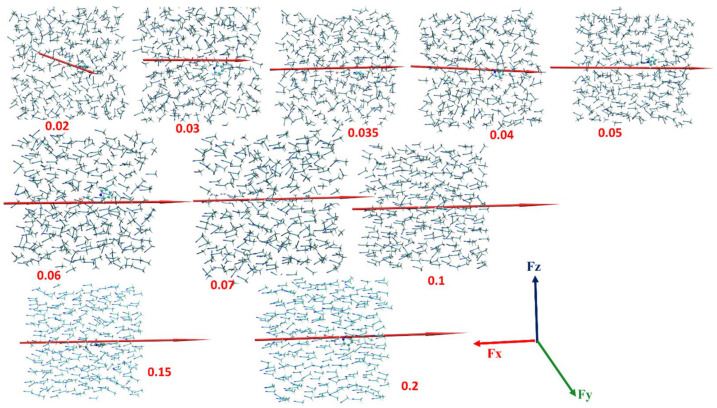
Snapshots from molecular dynamics (MD) simulations of a CH_3_CN solvent box at different positive values of electrical field **F_X_** values (in V/Å), noted in red below (or next to) each individual snapshot. The directions of the global dipole moment of the solvent ensemble in the box (in Debye units) are shown by the red arrows; the convention for the dipole moment vector assigns the head of the arrow as the positive pole. Reproduced from [77]: Copyright (2020), with permission from American Chemical Society with CC-BY license.

As an example, some snapshots in the MD trajectories of the dipole moments and global dipole moments (red arrows) for different $F_{x}$ values are shown in Figure 5. As seen, the solvent alignment increases with an increasing strength of $F_{x}$ while the global dipole moments gradually align with the field along the X axis. At around $F_{x}\;\geq \;0.15\;V/Å$ almost all CH_3_CN molecules and the global dipole moment are aligned.

Dubey et al. [77] note that the EEFs induce ordering and reorientation in a liquid phase, which is in agreement with previous experimental and computational results by Evans [78,79] and Cassone et al. [80]. While the EEF strength rises, the net dipole moment of the system is measured and at the same time the evolution of the entropy of the solvent is calculated [77]. It is seen that the increase of the strength of the applied EEF leads to increase of the order and the global dipole moment of the ensemble. This initial rise is followed by a levelling off for both the solvent’s entropy and dipole moment.

The overall picture that emerges from the published papers allows us to summarize the role of EF as follows. Theoretical calculations show that in the absence of EEF, solvents gradually assume a catalytic function consisting in the formation of an oriented internal electric field, facilitating “electron flow” associated with the transformation of reactants to the products. The application of OEEF leads to organization of the dipole moments of the solvent, i.e., the solvent molecules’ dipole moments orientation is gradually aligned to the orientation of applied field, and their resultant field is proportional to the strength of the applied external field. The collective organization has a large influence on the “catalyzing capabilities”, or proton motion, provided by the solvent. The effects of such a behavior are twofold. On the one hand, it restricts the positioning of the solvent molecules around the reactant cavity, thus suppressing the appearance of local uniformly oriented fields induced by the interaction of the solvent and the reactant. On the other hand, it causes formation of a global electric field, with a direction opposite to the direction of the applied EEF. The combination of these two intertwining effects results in (partial or full) screening of the OEEF, the degree of screening being proportional to the polarity/polarizability of the solvent. Although the applied OEEF is weakened by the reaction of the solvent environment, being different for each of the solvents, it is found that the catalytic ability inevitably appears once the OEEF value exceeds the self-organizing solvent field value.

In the last decade, many works have been published on the study of the transient absorption (TA) of various compounds, including of tautomers [81,82,83,84]. According to theory of TA, when a photoproduct is formed under excitation, the newly formed molecule interacts with the probe pulse as well and the new product absorption can be detected by a positive signal in the transient curve. Therefore, such behavior of the TA value should be increased at tautomers, investigated with short laser pulses, because of stimulating the transition from one to another tautomeric form, due to the condition in the observed reactant volume (for example—induced polarization, increased value of strength of EF etc.).

According to these studies, the strength of the electromagnetic field, and in particular of the EF, induced in the environment by the used femtosecond lasers, is very large. Therefore, the obtained results should also be interpreted from this point of view. Previously, in some experiments and theoretical studies, the aligning interaction was assumed to be due to a polarized electromagnetic wave interacting with the anisotropic molecular polarizability [85]. The author shows that the orienting interaction comes by exposing the molecule to a superimposed electrostatic field, based on the combination of a static electric field with a non-resonant optical field.

Combining results of the ab initio quantum chemical calculations and on-the-fly dynamics simulations of the tautomeric properties of salicylidene aniline, exposed to an EEF, Sobolewski and co-authors [86] made several important conclusions: the relative energy of the enol and keto tautomers can be controlled by applying the electric field along the main molecule axis; for a sufficiently strong electric fields, the enol-to-keto ratio can be changed and PT can be observed.

As concluded in [85,87,88], the strong induced dipole interaction, easily attainable with pulsed nonresonant femtosecond laser fields, can be used to enhance a weak permanent dipole orientation during the laser pulse. Under suitable conditions, a sizable orientation can persist even after the passage of the pulse. This should find application whenever molecular orientation is desirable and should be considered in all schemes that make use of combined electrostatic and pulsed radiative fields.

The explanation of this possibility gives a solution to the Schrödinger equation when only two eigenstates of energy are essential, corresponding to the values of the energy operator $E_{1}$ and $E_{2}$. It turns out that there are only two constant times—the constant $T_{1}$ for the diagonal elements of the density matrix and $T_{2}$—time constant for the non-diagonal elements of the matrix. The constant $T_{1}$ is called time of longitudinal, spin-lattice or dipole-lattice relaxation [89]. It corresponds to time during which the system changes from excited, Frank-Condon state to a state in equilibrium with the surrounding environment. The constant $T_{2}$ is called time of transverse, dipole-dipole or spin-spin relaxation. In principle, $T_{2}$ is in the picosecond-time scale, and it is always less than $T_{1}$, the value of which is in the nanoseconds area. Therefore, at least for several hundred picoseconds, the time behavior of the observed absorbance dependence is influenced only by the effect induced by the laser pump pulse value of EF in the reactant medium.

The effects of intense laser fields in the near- and infra-red (IR) region on chemical reactions was described by C. F. Matta et al. in [5,90,91]. They conclude that, to observe a concrete reaction, the effective potential of the system ($F_{\mathrm{ext}}$– $F_{\mathrm{int}}$) can be achieved by intensity of a plane polarized beam and “phase” between two laser pulses with different wavelengths. The strength of EEF is approximated as a sum of the field-free potential, a linear phase dependent field-dipole term, and a quadratic phase dependent field-polarizability term. Peaks in the dipole moment and polarizability of the system are found near to the transition states [5], so that the effects of the field are maximized near this point in the potential energy surface. The phase and the intensity of the external laser field can be adjusted to eliminate or even invert the potential energy barrier converting a transition state into a bound state.

In [92,93], the authors propose a model Hamiltonian analysis, which is two-fold. On one hand, they intend to propose a simple and yet general approach with the main aim of predicting the tautomeric behavior of molecular systems immersed in external electrostatic fields of various origin, on the basis of analysis of permanent and induced molecular multipole moments. On the other hand, they also aim to propose an approach that quantifies in a simple but rigorous manner the external electrostatic field influence on the proton transfer potential and the dynamics of PT process itself. A simple approach, based on analysis of permanent and induced molecular multipole moments, is proposed, with the main aim of predicting the tautomeric behavior of molecular systems immersed in external electrostatic fields of various origins. This approach also aims to quantifies in a simple, but rigorous, manner the external electrostatic field influence on the proton transfer potential and on the dynamics of proton transfer process itself, i.e., to investigate the tautomer equilibrium as a function of OEEF. As an example, the tautomeric equilibrium of 2-carbamido-1,3-indandione as a function of varying strength and directionality of electric fields was studied by using ab initio SCS-MP2 calculations. It was showed that the threshold value of the electric field capable of inversing the tautomeric equilibrium is established to be 0.0003 a.u. (0.1543 V/Å). It should be noted logically that such a value is individual for each tautomeric pair.

Cassone et al. [94] theoretically demonstrated the possibility of synthesizing hydrogen from liquid ethanol by means of intense electric fields at room temperature and in the absence of any catalyst or template. They suggested that by atomistically and electronically tracing the behavior of such system (under the influence of the EEF), the peculiar molecular arrangements are directly responsible for the $H_{2}$ production. Their calculations show that under intense EFs, an ethanol aqueous solution is able to maximize its entropy. This can be explained by the sustaining of an efficient protonic conduction through correlated proton transfers along a diffuse H-bond network or an anhydrous ethanol. At these conditions the intermolecular synergism is sizably less pronounced and it maximizes its entropy by creating hydrogen molecules.

## 4. How to Investigate ESIPT

As shown by theoretical and experimental investigations [11,12], in some cases, the tautomeric forms are near in energy (i.e., existing as an equilibrium tautomeric mixture) and the barriers between them low enough to provide fast and easy transition. The application of an EEF with an appropriate magnitude and direction could change the tautomeric equilibrium in the desired direction, that is, EF could be used as a tool to yield a specific tautomer with desired properties. In spite of the exciting concept for easy change of the tautomeric equilibrium by applying EEF, the real studies of tautomeric systems at these conditions are scarce.

This situation should be explained by the problems related to the values of EF strength, as suggested by the theoretical investigations, needed to observe the influence of EF on both phenomena (yield of a specific tautomer). For example, the values of EF strength, as predicted by the theoretical investigations, needed to obtain influence on a tautomeric equilibrium, are very high, varying in the range $0.001–0.01\;a.u.\;\left(0.001\;a.u.=0.05142\;V/Å=0.5142\;V/\mathrm{nm}\;=5.142.10^{6}\;V/\mathrm{cm};\;1\;a.u.=5.142.10^{11}\;V/m\right)$. This leads to a logical question: at which of the typical spectral methods (absorption, fluorescence etc.) such conditions are feasible in order to observe the influence of the magnitude, direction and duration of the applied electric field. Our opinion is that such behavior can be observed in experiments for measurements of the transient absorption in the femtosecond region at compounds with inherent tautomeric effect. In these experiments the used pump laser pulses have pulse duration of ~100 fs with diameter of pumping laser beam ~100 µm and pulse energy ~1 mJ, which leads to power intensity $I_{p}$ up to the $10^{14}\;W/{\mathrm{cm}}^{2}$. Therefore, due to the relation ($I_{p}\;\sim \;E^{2}$ [89]) between the power intensity and strength of EF (E)*,* at such conditions of the experiments, the strength of EF in investigated volume can achieve values up to $10^{7}–10^{8}\;V/\mathrm{cm}\;\left(0.1–1\;V/Å\right)$. The authors in [36] also reached a similar conclusion, giving the following relation for conversion:

(5) $$1\;V.{\;Å}^{-1}\;\approx \;10^{13}\;W.{\;\mathrm{cm}}^{-2}\;$$

**Figure 6:**
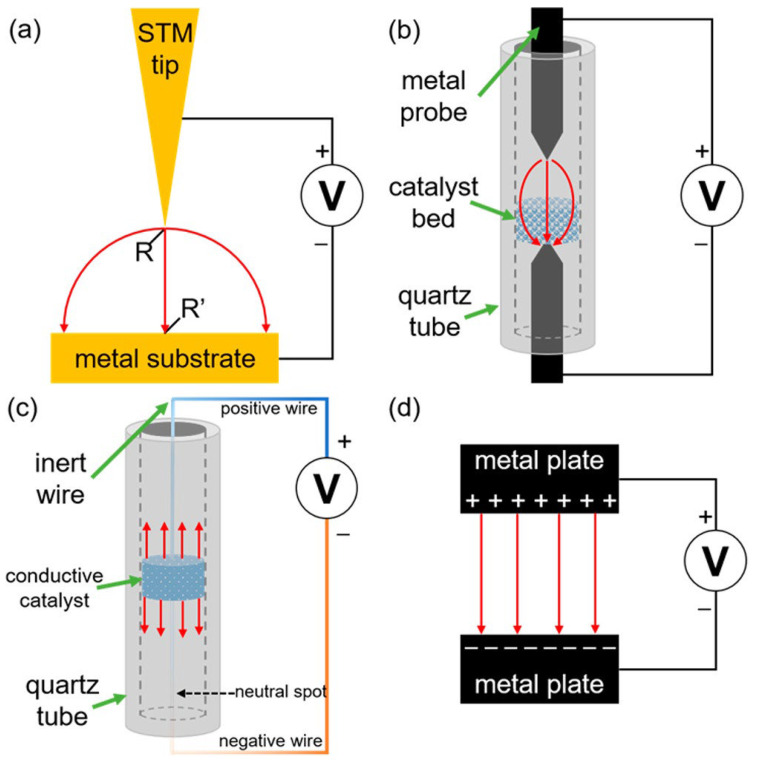
The four reactor types for investigations of EEF influence on processes: (**a**) scanning tunnelling microscope (STM) probe “nanoreactors”; (**b**) probe−bed−probe (PBP) reactors, in which a catalyst bed is placed in the gap between two probes; (**c**) continuous circuit (CC) reactors, in which the catalyst bed is integrated into an electric circuit (the surface charge gradient is represented with a color gradient in the wire) and (**d**) capacitor−type reactors. Red arrows indicate the general field structure; R and R′ denote adsorbed reactants. Reprinted with permission from [59]: Copyright 2018 American Chemical Society.

The effects of non-electrochemical activation of chemical reactions by using EEF have been exanimated as well. In 2018, Che et al. [59] distinguished between internal and external electric fields in the absence or presence of an applied electrical potential. They defined an EEF by utilization of an external power source to create the required surface electric field over the catalytically (or tautomerization) active zone, mostly metal surface. In their study different empirical approaches have been considered, namely (Figure 6) the use of scanning tunneling microscope (STM) probe nanoreactors, probe−bed−probe (PBP) reactors, integrated circuit reactors or capacitor reactors.

It is almost impossible to create an ideal experimental system, which is modelled using theory and successfully scaled to realistic experimental conditions. Another problem is to define the surface electric fields generated in these reactions due to the complexity of the real interaction conditions, for example catalyst surface geometries, and the lack of experimental measurement techniques. These factors lead to a complicated comparison between the practical reactor and the virtual one. Therefore, the most ideal case for both surfaces and the behavior of the electric field has been applied in order to explain the observed theoretical and experimental results. Some facts should be considered in this discussion: the fields cannot be defined without respect to the surface and the strength of the field cannot be measured directly. Therefore, for any future investigations, it would be more suitable to use physical models for estimation of these factors. The presence of some small defects, the addition of micro- or nanoparticles, and pore structures make the experiments even more complex, but they are also thought to increase the field strength. Field enhancement effects are well-known to occur around very small irregularities. In fact, this is an explanation for how the STM tip is able to generate such high electric fields with such a low applied voltage. Therefore, in the approaches to model theoretically, the use of each of the presented reactors and the comparison of theoretical with experimental data, the differences in the results due to the presence of defects, other particles and molecules and pores on the electrodes should be considered.

In ref. [95], Fried et al., by using IR spectroscopy, detected a single internal proton transfer in an entire protein. This method, by use of specific IR probes, is a combination of spectral separation and the ability to differentiate, in the time scale, the two protonation states in the equilibrium. The data demonstrate unambiguously that two protonation states of an intermediate analogue coexist in the active site of ketosteroid isomerase (KSI). The latter, in the context of a thermodynamic cycle, implies that the maximum thermodynamic advantage that KSI could capture from a concerted mechanism in which energy change is only 0.5 kcal.mol^−1^. The authors suggest that enzymes may not need to employ concerted acid–base mechanisms to catalyze difficult proton-transfer reactions. When an applied electric field is aligned in such a way as to stabilize electrostatically the formally covalent species, the degree of resonance increases, resulting in the overall stabilization of the molecule or transition state. This means that it should be possible to manipulate the kinetics and thermodynamics of non-redox processes using an external electric field if the orientation of the approaching reactants with respect to the field stimulus can be controlled, as Aragones et al. discussed in their study [96]. They provided experimental evidence that the formation of carbon–carbon bonds is accelerated by an electric field. A surface model system to probe the Diels–Alder reaction and coupled with a scanning tunnelling microscopy break-junction approach was designed. The technique, performed at the single-molecule level, is perfectly suited to deliver an electric-field stimulus across approaching reactants. As a result, fivefold increase in the frequency of formation of single-molecule junctions was found, resulting from the reaction that occurs when the electric field is present and aligned to favor electron flow from the dienophile to the diene.

Došlić et al. [97] investigated laser control on proton dynamics in the medium-to-strong intramolecular hydrogen bond of picolinic acid N-oxide (PANO). They extracted a 2D model potential from the DFT calculations that includes the proton transfer motion and the heavy atoms mode. The effects of the environmental degrees of freedom by means of their spectral density within the density matrix formalism was treated. The proton dynamics was monitored over time by calculating the nonlinear optical response nonperturbatively in the driving field. The approximations to dissipative dynamics led to two problems: first, with the phase in the pump-probe simulation, which can be overcome by introducing a carrier wave expansion of the reduced density matrix, thus allowing for the differentiation of various spectroscopic techniques. Secondly, the more problematic approximation is the perturbative treatment of most intramolecular degrees of freedom. They intended to overcome it by enlarging the quantum system by inclusion of the in-plane hydrogen bending mode and treating the remaining intramolecular degrees of freedom and solvent explicitly using mixed quantum-classical simulation.

## 5. Conclusions and Outlooks

Overall, the analyses performed on the basis of the published papers provide a clear and illustrative interpretation of the behavior of the solution and reacting compounds in the presence of EEF and show that EEF-mediated interaction processes (tautomerization, ESIPT, catalysis) in principle should be feasible in the solvent environment.

As far as the study of tautomeric compounds under the influence of EEF, as we noted, they are determined by the possibility of multivariate interactions in the solvents. It is important in the experiments to use the low values of the strength of the applied EFF in order not to induce an electrical breakdown in the studied volume. Our opinion is that the optimal scheme for such studies would resemble the following method. Initially, EF is applied, which does not cause a breakthrough in the medium, so that the corresponding orientation of the dipole moments of the solvent is realized. After that, a light field with a certain polarization is applied, considering the characteristic directions of influence of this field on the investigated processes (direction of the transition, direction of the dipole moment etc.) with intensity in the diapason 0.001–1 V/Å in accordance with theoretical predictions. The energy of the photon of this field should be such that it does not cause a $S_{0}$–$S_{1}$ transition, i.e., in many cases wavelength above 1000 nm (or less than 1 eV). The last step is the impact of the investigated process with the corresponding wavelength, allowing the determination of the absorption and emission properties of the investigated compound under the upper conditions. These would probably be part of the necessary conditions for the objective study of the influence of EF on the processes in tautomeric compounds, which are interesting from a practical point of view.

Therefore, EEF-mediated interaction processes will be better governed by the value of the homogeneous EF, especially for reactions in nonpolar and weakly polar solvents, and its direction with respect to the dipole moments and the axis along at which the investigated reaction takes place. These conclusions and the possible manifestations of the ability of OEEF to influence the physicochemical parameters of the interactions in various studied reactions give a ground to claim that this influence can be generalized for many other of the chemical reactions, including tautomerization processes. This opens possibilities for the practical realization of many of the theoretically predicted applications of tautomeric compounds.
